# Integrated extraction, structural elucidation and hypoglycemic mechanism of *Eucommia ulmoides* polysaccharides: a mini review

**DOI:** 10.3389/fphar.2025.1649040

**Published:** 2025-09-16

**Authors:** Ping Huang, Junbo Liu, Wencheng Mao, Hongmei Chen

**Affiliations:** ^1^ Hangzhou TCM Hospital Affiliated to Zhejiang Chinese Medical University, Hangzhou, China; ^2^ Hangzhou Academy of Agricultural Sciences, Hangzhou, China; ^3^ Shangrao Guangxin District People’s Hospital, Shangrao, China

**Keywords:** eucommia ulmoides polysaccharides, extraction, purification, structural characteristics, hypoglycemic activity, mechanism

## Abstract

The species *Eucommia ulmoides* Oliv. (EU) is gaining increasing attention from nutrition experts and health-conscious consumers due to its nutrient-providing properties. EU has been selected for inclusion in China’s Medicinal Food Directory because of its high safety profile. Polysaccharides are considered the main functional component and active ingredients of this plant. Modern pharmacological studies demonstrate that these polysaccharides, as primary bio-active components of EU, exhibit multiple bio-activities including effectiveness in relieving insulin resistance in diabetes models, lowering blood sugar, and improving diabetes complication. Diabetes represents an increasingly severe global metabolic epidemic that affects millions of people’s quality of life. Additionally, variations in extraction, isolation, and purification methods significantly impact the content, purity, and structural characterization of EU polysaccharides (EUP), thereby influencing its biological activity. Therefore, the present study reviewed the latest progress in the extraction, isolation, and purification methods, structural characteristics, and potential mechanisms of EUP based on a comprehensive literature search and compilation, aiming to provide a theoretical basis for in-depth research and product development.

## 1 Introduction

Diabetes has become one of the fastest-growing metabolic diseases globally, posing significant challenges to individual quality of life and socioeconomic burdens (Liu et al., 2022). Characterized by hyperglycemia, hyperlipidemia, and insulin resistance, it causes multi-organ damage, including kidneys, nerves, eyes, and heart, leading to complications such as blindness, stroke, and lower limb amputation (Bailey, 2000; DeFronzo et al., 2015). Global diabetes prevalence reached approximately 500 million cases in 2019, and this number is projected to increase by 51% by 2045 (Saeedi et al., 2019). China, the most populous in having highest number of diabetic patients, reported over 114 million diabetes patients in 2017, representing nearly 10% of its adult population (Zhang et al., 2022). Urgent preventive measures and novel therapeutics are therefore imperative. Research on natural products for diabetes intervention has expanded significantly, with polysaccharides emerging as key candidates due to their structural diversity, low toxicity, and synergistic effects with conventional therapies (Gao et al., 2024; Tian et al., 2024). These compounds enhance treatment efficacy while reducing adverse effects associated with standard anti-diabetic drugs.

*Eucommia ulmoides* Oliv. (EU), the sole species of the monotypic family Eucommiaceae, is a deciduous tree endemic to China and recognized as a premier medicinal resource (“plant gold”) (Bao et al., 2024). Classified as a superior-grade (shangpin) herb in Shennong Bencao Jing (神农本草经), it is documented to “tonify the center, boost qi, strengthen tendons and bones, and promote longevity with prolonged use” (Liu et al., 2020). With a 2,000-year history in traditional Chinese medicine (TCM), its medicinal significance is well-established (Zhao X. et al., 2024). To date, over 200 compounds have been isolated from EU, primarily including lignans (e.g., pinoresinol diglucoside), polysaccharides, iridoids (e.g., geniposidic acid), flavonoids, phenylpropanoids, triterpenes, and antifungal proteins (Bao et al., 2024; Liu et al., 2020; Zhang and Li, 2017). Among these, EU polysaccharides (EUP) emerges as a core bio-active component, demonstrating glycemic regulation, anti-inflammatory and antioxidant effects, immunomodulation, bone metabolism regulation, hepatoprotection and neuroprotection (Bao et al., 2024; Zhao X. et al., 2024; Huang et al., 2021).

In recent years, due to the potential medicinal value of EUP, extensive exploration and research have been conducted by scholars both domestically and internationally, yielding significant progress (Bao et al., 2024). However, studies on the preventive and therapeutic effects of EUP on diabetes-related diseases and their underlying mechanisms remain in the early stages, with limited clinical research. Although general pharmacology and phytochemistry of EU have been reported (Huang et al., 2021; He et al., 2014), existing studies lack assessment of extraction, structural elucidation and hypoglycemic mechanism of EUP. Therefore, this article examines the structural characteristics and pharmacological effects of EUP, by synthesizing existing research progress, proposes future research directions to provide a scientific foundation for the development and clinical application of EUP.

## 2 Extraction, isolation and purification methods

**FIGURE 1:**
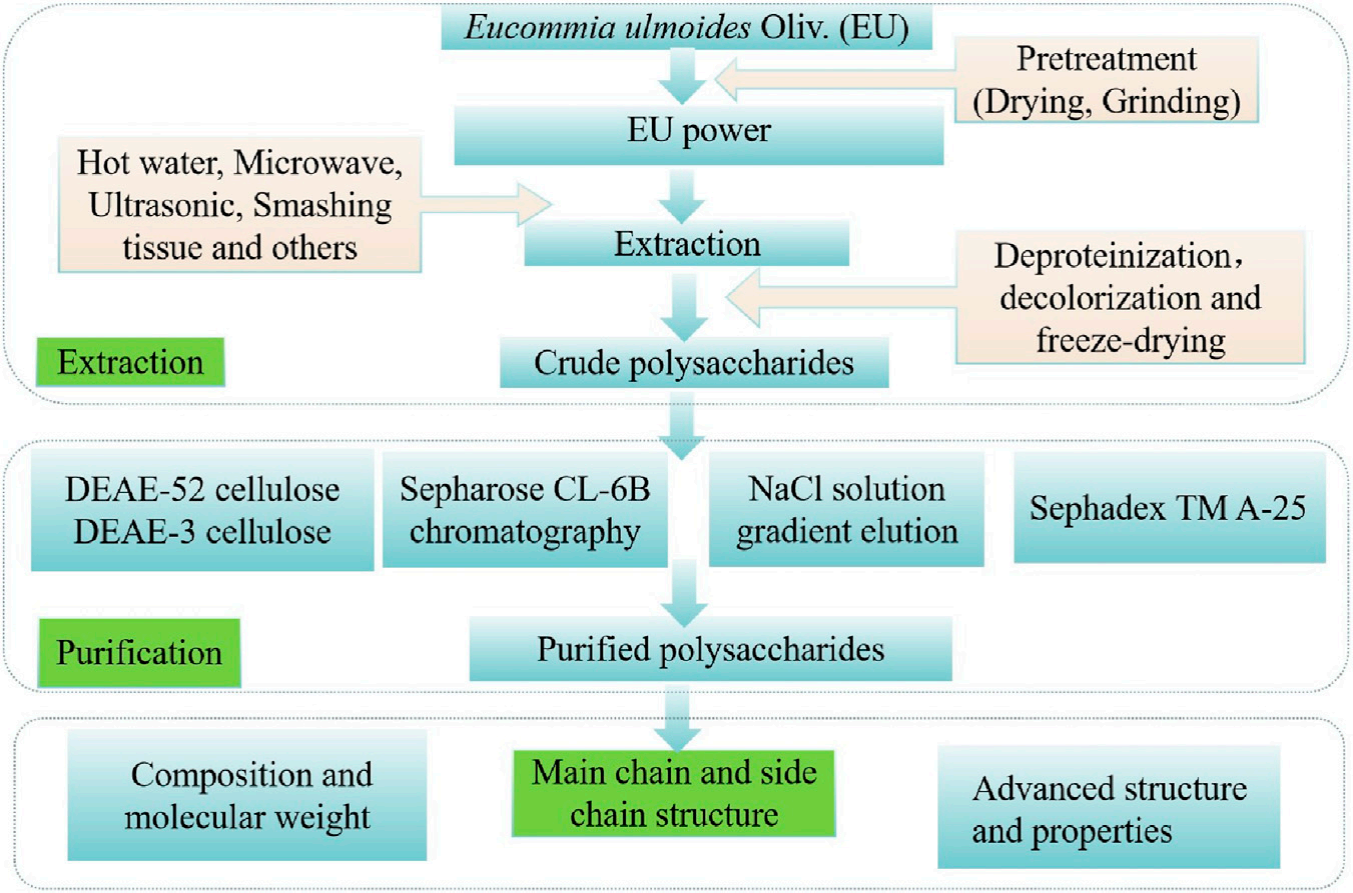
Extraction and purification methods, and structural features analysis of EUP.

Polysaccharides are one of the important active substances from EU. The yield improvement of EUP has become a research focus, driven by its validated applications in functional food development and therapeutic interventions. The procedures of extraction, isolation, and purification may affect polysaccharides yields. The flowchart for EUP extraction and purification is summarized in Figure 1.

### 2.1 Extraction of EUP

In the process of polysaccharide extraction, the selection of an appropriate pretreatment method is crucial. Treatment with ethanol, methanol, acetone and/or chloroform can be employed to remove lipophilic substances. Different extraction methods should be selected according to the physicochemical properties of target polysaccharides. Currently, common methods for polysaccharide extraction from EU include hot water extraction, ultrasonic-assisted extraction, and microwave-assisted extraction. Key parameters such as solid-liquid ratio, temperature, extraction time, extraction medium, ultrasonic power, and microwave power can significantly influence polysaccharide yields.

#### 2.1.1 Hot water extraction

Hot water extraction is currently the most commonly used method for extracting polysaccharides from TCM, primarily due to its simplicity, convenience, low cost and accessibility. In one study, the extraction yield of EUP was optimized, with optimal conditions identified as extraction time of 80 min, water-to-raw material ratio of 3:1, and three extraction cycles (Hong et al., 2013). Yan et al. (2023) investigated the effects of solid-liquid ratio, extraction time, number of extractions, and ethanol concentration on EUP yield, they determined the optimal process as solid-liquid ratio of 1:20 g/mL, extraction time of 3 h, three extraction cycles, ethanol concentration of 60%, yielding 4.79%. Zhu et al. (2022) optimized extraction conditions as follows: solid-liquid ratio of 1:60 (g: mL), extraction time of 40 min, ethanol concentration of 60%, and a yield of 44.53%. Qi and Zhou (2011) adopted uniform design and multiple regression analysis method to optimize the EUP extraction process, resulting in optimal parameters of solid-liquid ratio 1:5 g/mL, extraction temperature 100 °C, extraction time of 7 h, and a maximum yield of 8.231%. Zeng et al. (2018) optimized polysaccharide extraction from EU leaf tea, establishing optimal conditions as solid-liquid ratio 1:25 g/mL, extraction time of 1.5 h, extraction temperature 51 °C, and a yield of 8.48%.

#### 2.1.2 Ultrasound-assisted extraction method

To overcome the issue of long extraction time for hot water, ultrasonic-assisted technology accelerates the dissolution of polysaccharides through cavitation effect. Yang et al. (2019) optimized and obtained polysaccharides extraction parameters from EU, the optimal conditions were determines as solid-liquid ratio of 1:30 g/mL, ultrasound temperature 50 °C, ultrasound power 400 W, and ultrasound time 30 min. The average polysaccharides yield from EU bark was 2.16%. Xia and Pu (2019) employed orthogonal design to optimize ultrasonic extraction of EUP. Results showed that under conditions of ultrasonic power 250 W, extraction temperature 80 °C, extraction time 40 min, solid-liquid ratio 1:35 g/mL, and two extraction cycles, the EUP yield researched 4.89%. In order to enhance polysaccharides extraction efficiency from EU leaves, Chen et al. (Chen and Yang, 2020) utilized an ultrasound-enzyme synergistic method and optimized the process. Plackett-Burman screening identified pH, ultrasonic power, and composite enzyme dosage as primary factors affecting yield. Box-Behnken optimization revealed the optimal conditions: composite enzyme dosage 3.7% (w/w), pH 4.0, ultrasonic power 100 W, extraction time 15 min, temperature 45 °C, and solid-liquid ratio 1:20 g/mL. The achieved polysaccharide yield was 4.79%, closely matching the theoretical yield of 4.87%.

#### 2.1.3 Microwave-assisted and flash extraction method

To further efficiency and energy saving, microwave and flash extraction achieve instantaneous penetration through electromagnetic fields/mechanical shear forces. Microwave-assisted extraction (MAE) significantly shortened extraction time and improved product yield. Ji et al. (2021) obtained the optimal extraction conditions were microwave power 640 W, microwave time 90 s, NaOH concertration 3% (w/v), and soaking time 90 min, achieving an acidic polysaccharide yield of 6.35%. This process enhanced operational convenience and resource utilization efficiency. Xu et al. (2018) employed MAE to extract polysaccharides from EU leaves. Under conditions of 74 °C, solid-liquid ratio 1:29 g/mL, and extraction time 15 min, the polysaccharide yield reached 12.31%, closely matching predicted values and representing a 2.9-fold increase over traditional hot reflux extraction. Chen et al. (2023) studied ultrasound-microwave synergistic extraction for EU leaf polysaccharides. Plackett-Burman screening identified solid-liquid ratio, extraction time, and temperature as key factors. Box-Behnken optimization determined optimal conditions: solid-liquid ratio 1:30 g/mL, ultrasonic power 130 W, temperature 49 °C, microwave power 200 W, and extraction time 20 min. The actual yield was 4.02%, approximating the theoretical yield of 4.08%. Researchers have employed alternative techniques for polysaccharide extraction from EU. Qi et al. (Qi et al., 2020) utilized flash extraction for EU leaves, optimizing parameters including extraction cycles, voltage, and solid-liquid ratio. Optimal conditions were determined as solid-liquid ratio 1:30 g/mL, voltage 160 V, extraction time 60 s, and two extraction cycles, yielding 3.36%.

#### 2.1.4 Alkaline treatment and membrane filtration

Alkaline treatment and membrane separation technology significantly enhances the yield and purity of bio-active polysaccharides, while reducing energy consumption and environmental impact. Zheng et al. (2023) optimized alkaline treatment extraction (NaOH concentration 0.1–0.5 M, 60 °C–90 °C) to isolate polysaccharides from EU leaves, achieving a 12.3% yield with enhanced immunomodulatory activity while identifying high energy consumption as a key environmental drawback. While, EUP were extracted using membrane filtration technology, specifically ultra-filtration with molecular weight cut-off (MWCO) membranes of 30 kDa and 10 kDa. Results showed that the ultra-filtration significantly reduced protein content, enhanced polysaccharide homogeneity, and yielded fractions with varying molecular weights (ELP1, ELP2, and ELP3) exhibiting different antioxidant activities, with ELP3 demonstrating the highest scavenging efficacy against ˙OH and ABTS˙^+^ (Le et al., 2025).

To sum up, comparative analysis indicates that extraction method significantly influences EUP yield. Ultrasonic-assisted, microwave-assisted, flash, alkaline treatment and membrane filtration extraction typically yield less than hot water extraction. Current methods still exhibit relatively low efficiencies, necessitating advanced technologies for industrial-scale EUP production in functional foods and pharmaceuticals. Synergistic approaches combining multiple methods warrant further investigation. In summary, hot water extraction, due to its simple process, easy operation, low cost, and high yield, is suitable for future large-scale EUP production.

### 2.2 Isolation and purification of EUP

Crude polysaccharides from EU obtained by conventional extraction contain significant impurities (e.g., pigments, proteins, and inorganic salts), which interfere with structural characterization and bioactivity studies. Thus, sequential degreasing, decolorization, deproteinization, and fractionation are required. Deproteinization methods include sevage method (a commonly used technique for the isolation and purification of polysaccharides, achieving purification by removing protein impurities), trichloroacetic acid (TCA) method (one of the most popular methods for protein removal from samples), and hydrochloric acid (HCI) method (a class of chemical analysis methods using hydrochloric acid as the core reagent, primarily used for sample decomposition or concentration detection). Huang et al. (2014) reported deproteinization efficiencies of 95.76% (HCl), 93.23% (TCA), and 91.48% (Sevag), with polysaccharide retention rates of 30.44%, 40.29%, and 65.49%, respectively. Although HCl achieves the highest protein removal, it causes severe polysaccharide degradation. TCA and Sevag exhibit similar deproteinization efficiency, but Sevag preserves significantly more polysaccharides (65.49% vs. 40.29%), establishing it as the optimal method for EU leaf polysaccharides. Decolorization method primarily involve hydrogen peroxide (H_2_O_2_) oxidation and activated carbon adsorption. H_2_O_2_ demonstrates superior decolorization efficacy for EUP compared to activated carbon. Yan et al. (2023) combined Sevag deproteinization with Sephadex G-200 gel chromatography, yielding purified EUP with 89.12% total sugar, 2.03% protein, and 9.45% uronic acid. Yang et al. (2019) optimized activated carbon decolorization for EU bark polysaccharides via orthogonal design: 0.6% carbon dosage, 60 °C, 50 min, pH 5.0, achieving 76.20% decolorization and 62.68% polysaccharide retention. Integrated purification using macroporous resin (e.g., AB-8) simultaneously removes proteins and pigments while minimizing polysaccharide loss4. Zhang (2020) purified EUP under conditions: 0.6 mg/mL sample concentration, pH 6.0, 1.0 mL/min flow rate, elution with 150 mL of 65% ethanol. This increased polysaccharide mass fraction from 10.2% to 35.8% (3.5-fold) and enhanced anti-fatigue activity in animal models. For structural and functional studies, single-step purification is insufficient. To obtain EUP with uniform molecular weight and polarity, sequential chromatography is essential ion-exchange (e.g., DEAE-52 cellulose column with 0.1–0.3 mol/L NaCl gradient) and gel filtration (e.g., Sephadex G-100/G-200). This yields homogeneous polysaccharide components for downstream applications.

### 2.3 Structural features analysis of EUP

Polysaccharide structural analysis is critical as structural diversity directly determines bioactivity (Ai et al., 2023). This encompasses monosaccharide composition, molecular weight, glycosidic bond type, linkage patterns and higher-order conformations (Zhang et al., 2023).

#### 2.3.1 Composition and molecular weight

Monosaccharide composition of EUP is diverse, mainly composed of glucose (Glc), fructose (Fru), Mannose (Man), fucose (Fuc), Galactose (Gal) and Arabinose (Ara), with minor Xylose (Xyl), Rhamnose (Rha), Ribose (Rib), and Galacturonic acid (GalA). And the molecular weight range spans from 1.1 kDa to 1,653 kDa. Cui et al. (2023) isolated an immune enhancing polysaccharide (*E. ulmoides* leaf polysaccharide, ELP) from EU leaves, and found it contains Ara, GalA, Gal, and Xyl, and trace glucose. Song et al. (2023) separated an acid polysaccharide (EuOCP3) from the bark of EU. The monosaccharide composition and relative molecular weight were analyzed by using the 1-phenyl-3-methyl-5-pyrazolone pre-column derivatization method and gel permeation chromatography (GPC). EuOCP3, mainly consists of Ara, GalA, Rha, Gal, Glc, glucuronic acid, Man, and fucose, with a relative molecular weight of 3.81 × 10^4^ Da. Huang et al. (2014) purified an acid heteropolysaccharide EU polysaccharide-1 (EOP-1) from EU leaves, High performance gel permeation chromatography (HPGPC) analysis revealed a molecular weight was 60 kDa. Methylation-gas chromatography/mass spectrometry (GC/MS) analysis indicated that EOP-1 contains D-GalpA, D-Glcp, D-Galp, L-Araf, and L-Rhap residues. Chen et al. (2023) isolated a β-type acidic polysaccharide (molecular weight: 1,653 kDa) from leaves using ultrasonic degradation, Sevag method, and DEAE-52 cellulose chromatography. Its composition includes fructose, glucose, N-acetyl-D-glucosamine, galactose, and arabinose. Additionally, Zhang et al. (2011) obtained a heteropolysaccharide composed of L-rhamnose, D-fucose, D-arabinose, D-xylose, D-glucose, and D-galactose via hot water extraction, S-8 macroporous resin decolorization, Sevag method, and DEAE-52 cellulose chromatography. Cui et al. (2023) purified a heteropolysaccharide (arabinose, galacturonic acid, galactose, rhamnose, trace glucose) through ethanol extraction, DEAE-52 cellulose purification, and NaCl gradient elution. Song et al. (2023) extracted an acidic polysaccharide (molecular weight: 38.1 kDa) from bark using hot water extraction, Sevag method, DEAE-52 chromatography, and NaCl gradient elution. Wei et al. (2023) isolated a heteropolysaccharide (molecular weight: 25.1 kDa) from bark via hot water extraction, freeze-drying, DEAE-3 cellulose chromatography, and NaCl gradient elution, containing mannose, rhamnose, galacturonic acid, glucose, galactose, xylose, and arabinose. Li et al. (2017) purified a polysaccharide (molecular weight:1.1 kDa) from bark using hot water extraction and DEAE-52 chromatography, which contains the 2,3,4-Me_3_-Galp structure and comprises rhamnose, arabinose, galactose, mannose, and glucose.

#### 2.3.2 Main chain and side chain structure

Fourier transform infrared spectroscopy (FT-IR) and nuclear magnetic resonance (NMR) were used to determine the basic structure of the EuOCP3. The results showed that the main chain of EuOCP3 is composed of →4)-α- GalpA-(1→4)-α-GalpA-(1→,→4)-α-GalpA-(1→5)-α-Araf-(1→,→4)-α-GalpA-(1→2)-α-Rhap-(1→,→4)-α-GalpA-(1→5)-α-Araf-(1→2)-α-Rhap-(1→repeating fragment. On the side chains substituted at C-2 and C-5 positions of →2,3,5)-α-Alaf-(1→), there are residues of T-β-Alaf→ and T-β-Alaf→4) -GalpA- (1→) (Song et al., 2023). Methylation-gas chromatography/mass spectrometry (GC/MS) analysis indicated that EOP-1 contains D-GalpA, D-Glcp, D-Galp, L-Araf, and L-Rhap residues. Its backbone consists of →4)-α-D-GalpA-(1→, with side chains comprising 1,4-D-Galp (45.11%), 1,6-D-Galp (35.90%), 1,5-L-Araf (0.90%), and 1,2-L-Rhap (10.50%) (Huang et al., 2014). The polysaccharide contains the 1,4-D-GalpA backbone, characterzing it as a galacturonic acid polysaccharide. It features a→2,3,5)-α-Araf-(1→ structure with eight monosaccharides: arabinose, galacturonic acid, rhamnose, galactose, glucose, glucuronic acid, mannose, and fucose (Song et al., 2023).

#### 2.3.3 Advanced structure and properties

Le et al. (2025) conducted a preliminary morphological and structural analysis of polysaccharides from EU leaves using scanning electron microscopy and circular dichroism, and assessed the thermal stability via thermogravimetric analysis coupled with differential scanning calorimetry. Results revealed a dense, smooth, and coiled network structure, confirming an acidic polysaccharide with high purity and absence of triple helix conformation. This acidic polysaccharide exhibits good thermal stability below 200 °C.

In summary, only a limited number of polysaccharide types have been isolated and purified from EU medicinal materials to date, and their detailed structural characterization remian incomplete, necessitating further investigation. As summarized in Table 1, the current understanding of extraction methods, isolation and purification processes, and structural characteristics of *Eucommia ulmoides* polysaccharides is still limited.

**TABLE 1:** Structural characteristics of EUP.

Source	Extraction and purification method	Polysaccharide type	Major glucoside linkage	Monosaccharide composition	Molecular weight	Activity	Ref
Leaves of EU	Ultrasonic degradation method, Sevag method, DEAE-52 cellulose column chromatography	β-type acidic polysaccharides	—	Fructose, glucose, N-acetyl-D-glucosamine, galactose, and arabinose	1,653 kDa	Anti-coagulant	Chen et al. (2023)
Leaves of EU	Hot water extraction, Sevag deproteinization, hydrogen peroxide decolorization, SepharoseCL-6B chromatography	Galacturonic acid polysaccharides	1,4-D-GalpA	Galacturonic acid, galactose, rhamnose, mannose, arabinose, glucose	600 kDa	—	Huang et al. (2014)
Leaves of *EU*	Ethanol extraction, DEAE-52 cellulose column chromatography, NaCl solution gradient elution	Hetero-polysaccharides	—	Arabinose, galacturonic acid, galactose, rhamnose, and glucose	—	Immuno-modulation	Cui et al. (2023)
Bark of *EU*	Hot water extraction, Sevag method, DEAE-52 anion exchange column, NaCl solution gradient elution	Hetero-polysaccharides	→4)-α-GalpA-(1→4)-α-GalpA-(1→,→4)-α-GalpA-(1→5)-α-Araf-(1→,→4)-α-GalpA-(1→2)-α-Rhap-(1→,→4)-α-GalpA-(1→5)-α-Araf-(1→2)-α-Rhap-(1→	Arabinose, galacturonic acid, rhamnose, galactose, glucose, glucuronic acid, mannose, and fucose	38.1 kDa	Anti-osteoporosis	Song et al. (2023)
Leaves of *EU*	Hot water extraction method, S-8 macroporous resin decolorization method, Sevag method, DEAE-52 cellulose column chromatography	Hetero-polysaccharides	—	L-rhamnose, D-fucose, D-arabinose, D-xylose, D-glucose, and D-galactose	—	Complement- inhibitory	Zhang et al. (2011)
Bark of *EU*	Hot water extraction, freeze-drying, DEAE-3 cellulose column purification, NaCl gradient elution	Hetero-polysaccharides	—	Mannose, rhamnose, galacturonic acid, glucose, galactose, xylose, and arabinose	25.1 kDa	Ameliorate aging-associated gut dysbiosis	Wei et al. (2023)
Bark of *EU*	Extraction by hot water and purification by DEAE-52 column	Hetero-polysaccharides	2, 3, 4-Me3-Galp,→3,4-Rha–1→3-Glc-1→,→4-Man–1→ 4-Glc-1→,→4-Glc–1→4-Glc-1→,→4-Glc–1→3-Glc-1→,→3-Glc–1→4,3-Rha–1→,→3,4-Rha– 1→3,6-Gal-1→,→3,6-Gal–1→3,6-Gal–1→6-Gal-1→,→6-Gal–1→6-Gal–1→3-Gal-1→,Man–1→3, 6-Gal-1→	Rhamnose, arabinose, galactose, mannose, and glucose	1.1 kDa, 358.1 kDa	Anti-inflammatory	Li et al. (2017)
Bark of EU	Hot water reflux extraction, DEAE Sephadex TM A-25 cellulose Column purification	Hetero-polysaccharides	—	Glucose, fructose, mannose, fucose, galactose, arabinose	3.17 kDa	Osteo-immunomodulatory	Deng et al. (2019)
Bark of EU	Hot water reflux extraction, DEAE Sephadex TM A-25 cellulose Column purification	Hetero-polysaccharides	—	Rhamnose, arabinose, galactose, mannose, glucose	1,146.32 kDa	Immuno-enhancement	Feng et al. (2016)

## 3 Antidiabetic effects of EUP and their mechanisms

Diabetes mellitus is a metabolic disorder characterized by persistent hyperglycemia, primarily resulting from defective insulin secretion or impairment biological action. The two predominant forms are type 1 (T1DM) and type 2 diabetes mellitus (T2DM), with T1DM accounting for approximately 5% of cases and T2DM comprising about 95% (Jeremiah et al., 2024). The antidiabetic efficacy of EUP has been extensively validated, driving increased research focus on mechanistic elucidation. Nevertheless, comprehensive review addressing these mechanisms remain limited. This section details EUP’s modes of action through glucose metabolism regulation, pancreatic protection, oxidative stress mitigation, inflammation suppression, lipid metabolism improvement and gut microbiota modulation.

### 3.1 Regulation of glucose metabolism

**FIGURE 2:**
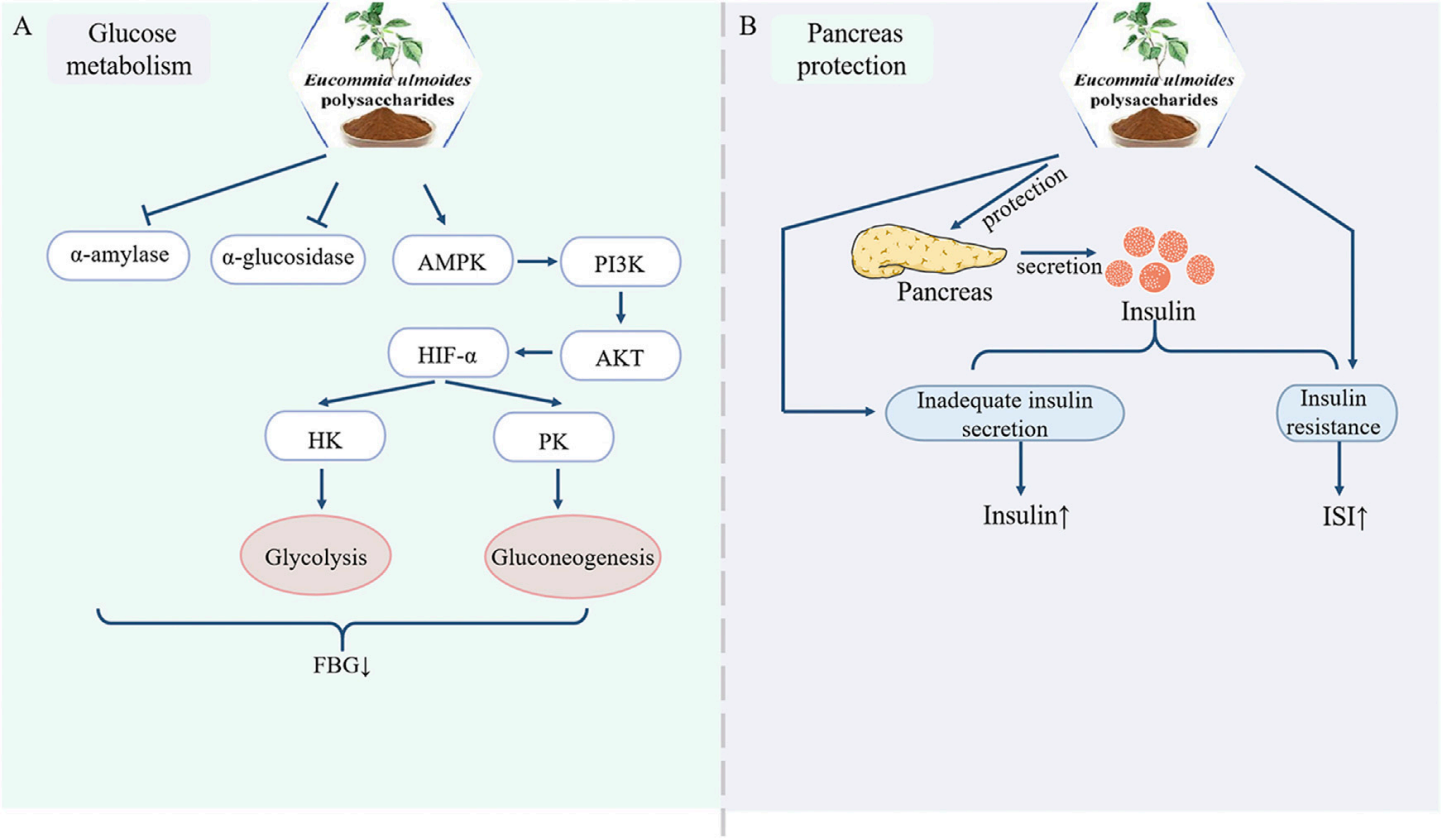
Pharmacological effects of EUP in the regulation of glucose metabolism and protection of the pancreas. **(A)** Regulation of glucose metabolism. **(B)** Protection of the pancreas.

Elevated blood glucose levels constitute a hallmarks of diabetes mellitus, which if unmanaged may precipitate severe complications. Current research confirms that EUP effectively reduce hyperglycemia and ameliorate glucose metabolism disorders. Since α-amylase and α-glucosidase catalyze dietary starch hydrolysis into glucose, suppressing their activity decelerates intestinal glucose absorption and delays glucose transport into the bloodstream, thereby lowering blood glucose levels. Lang et al. (2020) demonstrated that Eucommia ulmoides leaf polysaccharides exhibit dose-dependent inhibition of α-glucosidase activity, a finding extended by Gong et al. who confirmed EUP’s dual inhibitory effects on α-amylase and α-glucosidase. These enzymes catalyze dietary starch hydrolysis into glucose, and their suppression reduces intestinal glucose absorption rates (Gong et al., 2024). Furthermore, EUP modulates key glycolytic enzymes: restores diminished activities of hexokinase (HK) and pyruvate kinase (PK) in insulin-resistant HepG2 cells, and upregulates AMPK/PI3K/AKT pathway genes and enhances glycolysis via HIF-1α-mediated induction of glucose transporters (GLUTs), hexokinase (HK), and phosphofructokinase (PFK). *In vivo* validation showed significant reductions in fasting blood glucose (FBG) levels (>40% vs. controls) in streptozotocin (STZ, 50 mg/kg)-induced diabetic mice and tetracosactide-induced diabetic models (Su et al., 2016a; Liu et al., 2010). Collectively, EUP exerts anti-diabetic effect through coordinated regulation of glucose-metabolizing enzymes and signaling pathways (Figure 2A).

### 3.2 Protection of the pancreas

As a pivotal hormone synthesized by pancreatic β-cells, insulin plays a crucial role in glucose homeostasis regulation. Insufficient insulin secretion or insulin resistance (IR) compromises this regulation, leading to chronic hyperglycemia and metabolic dysregulation (Berbudi et al., 2025). In T1DM, insulin deficiency arises from autoimmune destruction of pancreatic β-cells (Moradi et al., 2025). In T2DM, IR manifests as diminished insulin responsiveness in target tissues (muscle, liver, adipose), disrupting glucose/lipid/protein metabolism (Liu et al., 2025). Modern pharmacological studies have demonstrated that EU leaves polysaccharides preserve pancreatic tissue by restricting the expression of Caspase-3, p38MAPK and TGF-1 (Lang et al., 2020). In T2DM model mice, characteristic pathological changes were observed, including pancreatic islet vacuolation, islet cell atrophy, structural deformation, and nuclear pyknosis. EUP treatment significantly ameliorated these pathological alterations, as evidenced by reduced vacuolization and attenuated cellular atrophy (Xu et al., 2020). Furthermore, while STZ-induced diabetic mice exhibited markedly decreased insulin content, EUP administration significantly elevated insulin levels (p < 0.01) and improved the insulin sensitivity index by 32% (Su et al., 2016a). These findings collectively indicate that EUP exerts protective effects against pancreatic dysfunction by addressing both insulin secretion deficiency and insulin resistance (Figure 2B).

### 3.3 Mitigation of oxidative stress

**FIGURE 3:**
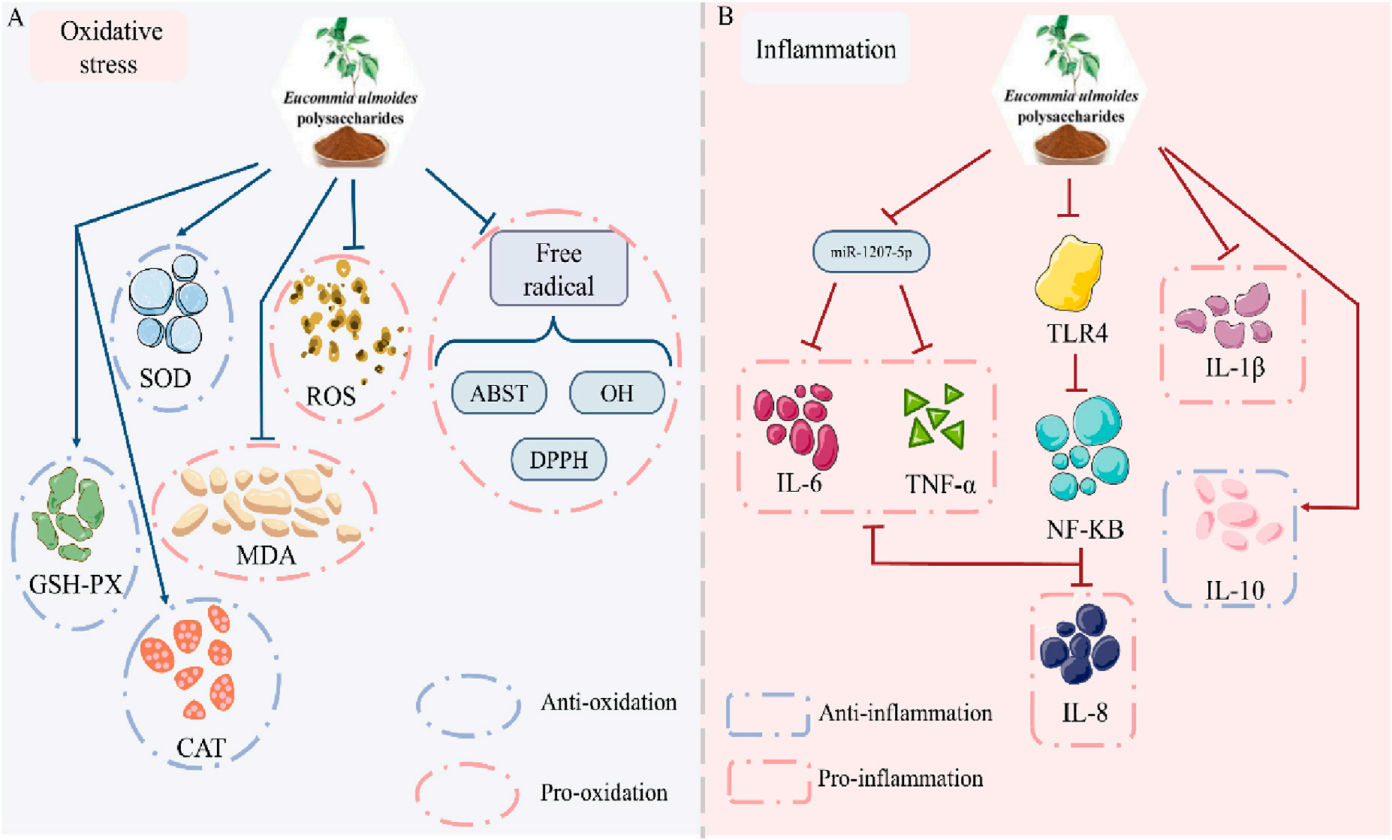
Pharmacological effects of EUP in the mitigation of oxidative stress and inhibition of inflammation. **(A)** Mitigation of oxidative stress. **(B)** Inhibition of inflammation.

Excessive hyperglycemia and reactive oxygen species (ROS) overproduction induce oxidative stress-mediated cellular damage, a key contributor to late-stage diabetic complications (Tonin et al., 2024; Dworzański et al., 2020). *In vitro* analyses reveal EUP’s potent radical-scavenging capacity: DPPH radicals, 87.05% clearance; ABTS radicals, 101.17% clearance; Hydroxyl radicals, 62.92% clearance, confirming significant antioxidant activity (Peng et al., 2024). In diabetic models, elevated ROS, hydroxyl radicals, and malondialdehyde (MDA) indicate pathological oxidative stress. EUP administration reduces pro-oxidants (ROS, hydroxyl radicals and MDA), and enhances antioxidant defense like superoxide dismutase (SOD), glutathione peroxidase (GSH-Px), and catalase (CAT) (Xu et al., 2020; Chen et al., 2020; Wang et al., 2016). This dual modulation-suppressing oxidant generation while boosting antioxidant capacity-demonstrates EUP’s efficacy in restoring redox homeostasis (Figure 3A).

### 3.4 Inhibition of inflammation

The crosstalk between inflammatory mediators and signaling pathways disrupts glucose homeostasis, contributing to diabetes pathogenesis (Su et al., 2020; Yang et al., 2022). The pro-inflammatory cytokines tumor necrosis factor α (TNF-α), interleukin-1β (IL-1β) and interleukin-6 (IL-6) induce cytokine production, creating an inflammatory cascade that amplifies inflammatory signals. Interleukin-8 (IL-8) acts as a chemokine that recruits neutrophils to inflammatory sites, thereby enhancing the inflammatory response. In STZ-induced diabetic mice, we observed a significant inflammatory response characterized by markedly elevated levels of TNF-α, IL-8, and IL-6. Notably, these cytokines activated intracellular inflammatory pathways through the TLR4 signaling pathway. In contrast, mice treated with EUP showed decreased levels of both TLR4 and NF-κB, which subsequently led to reduced levels of TNF-α, IL-8 and IL-6 (Su et al., 2016a). Additionally, EUP administration significantly lowered IL-8, IL-1β and IL-6 levels in db/db mice (Chen et al., 2020). *In vitro* experiments demonstrated that EUP inhibited high glucose-induced IL-6 and TNF-α production in HK-2 cells. Subsequent studies revealed that these polysaccharides downregulated miR-1207–5p expression, thereby suppressing inflammation (Shen et al., 2023). Besides, EUP was shown to promote expression of the anti-inflammatory cytokine interleukin-10 (IL-10) (Feng et al., 2016). Collectively, these findings demonstrate that EUP exerts potent anti-inflammatory effects by both upregulating IL-10 expression and downregulating pro-inflammatory factors including TNF-α, IL-8, IL-1β, and IL-6 (Figure 3B).

### 3.5 Improvement of lipid metabolism

**FIGURE 4:**
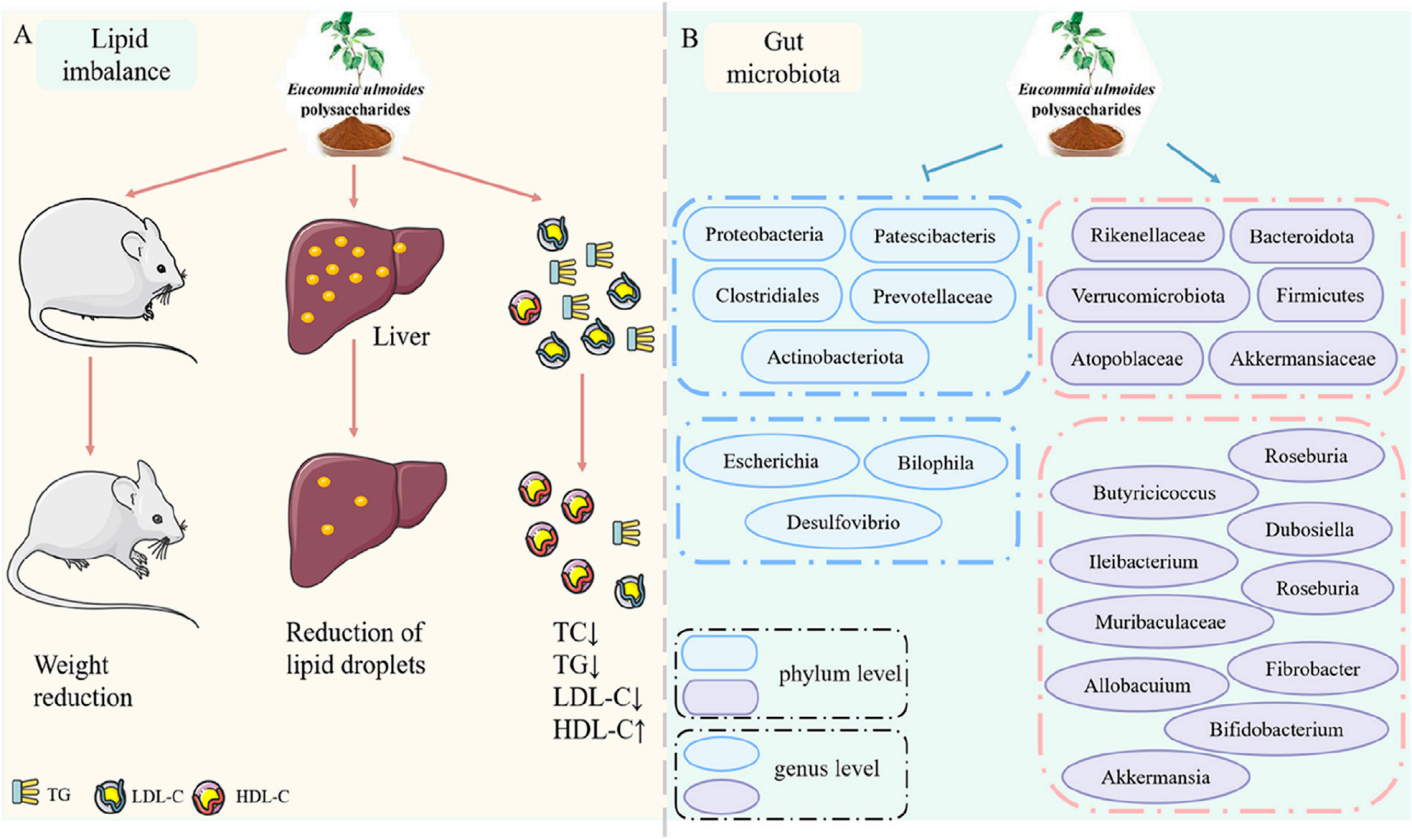
Pharmacological effects of EUP in the improvement of lipid metabolism and regulation of gut microbiota. **(A)** Improvement of lipid metabolism. **(B)** Regulation of gut microbiota.

In the early stages of diabetes, IR-induced excessive fat accumulation is frequently accompanied by lipid metabolism disorders, resulting in hyperlipidemia (Xu et al., 2024; Su et al., 2016b). Standard lipid profile analyses typically include measurements of total cholesterol (TC), triglycerides (TG), low-density lipoprotein cholesterol (LDL-C), and high-density lipoprotein cholesterol (HDL-C). Both db/db mice and STZ-induced diabetic models exhibited elevated TC, TG, and LDL-C levels along with reduced HDL-C, confirming diabetes-associated lipid metabolism dysregulation. EUP treatment effectively reversed these abnormal lipid profiles by Reducing TC (by 32%), TG (41%), and LDL-C (28%), and Increasing HDL-C levels (1.7-fold) (Su et al., 2016a; Chen et al., 2020). Beyond serum lipid parameters, hepatic lipid droplet accumulation and increased body weight in db/db mice further demonstrated lipid metabolic dysfunction. Notably, EUP administration decreased hepatic lipid content by 45%, reduced body weight gain by 23% (Chen et al., 2020). These findings collectively demonstrate that EUP significantly improves lipid metabolism disorders and effectively regulates blood lipid parameters (Figure 4A).

### 3.6 Regulation of gut microbiota

The Gut microbiota plays a vital role in maintaining physiological homeostasis (Su et al., 2020). Accumulating evidence suggests a correlation between gut microbiota dysbiosis and the development of diabetes-related complications (Yang et al., 2022; Brugman et al., 2006). Zhang et al. (2022) demonstrated that EUP alleviated gut microbiota dysbiosis by both reducing bacterial lipopolysaccharides release and inhibiting microglia-mediated TLR4/NF-κB/MAPK signaling pathways. Additionally, Wei et al. (2023) demonstrated that EUP reshaped gut microbial communities, inhibited ROS accumulation, and extended lifespan in fruit flies, suggesting their potential role in regulating gut microbiota. In obese diabetic mice, EUP treatment enriched short-chain fatty acid (SCFA)-producing bacteria, increased SCFA production by 2.3-fold, reduced endotoxin levels by 58%, upregulated occludin expression (1.8-fold), strengthening intestinal barrier integrity (Wang et al., 2023). Current limitations are insufficient clinical evidence for EUP’s microbiota-mediated antidiabetic effects and need for human trials to validate animal study findings. EUP exhibits multi-target microbiota-modulating effects by promoting beneficial bacterial growth (+39% *Lactobacillus*), suppressing pathogenic species (−62% Enterobacteriaceae), and restoring gut barrier function (Figure 4B).

**FIGURE 5:**
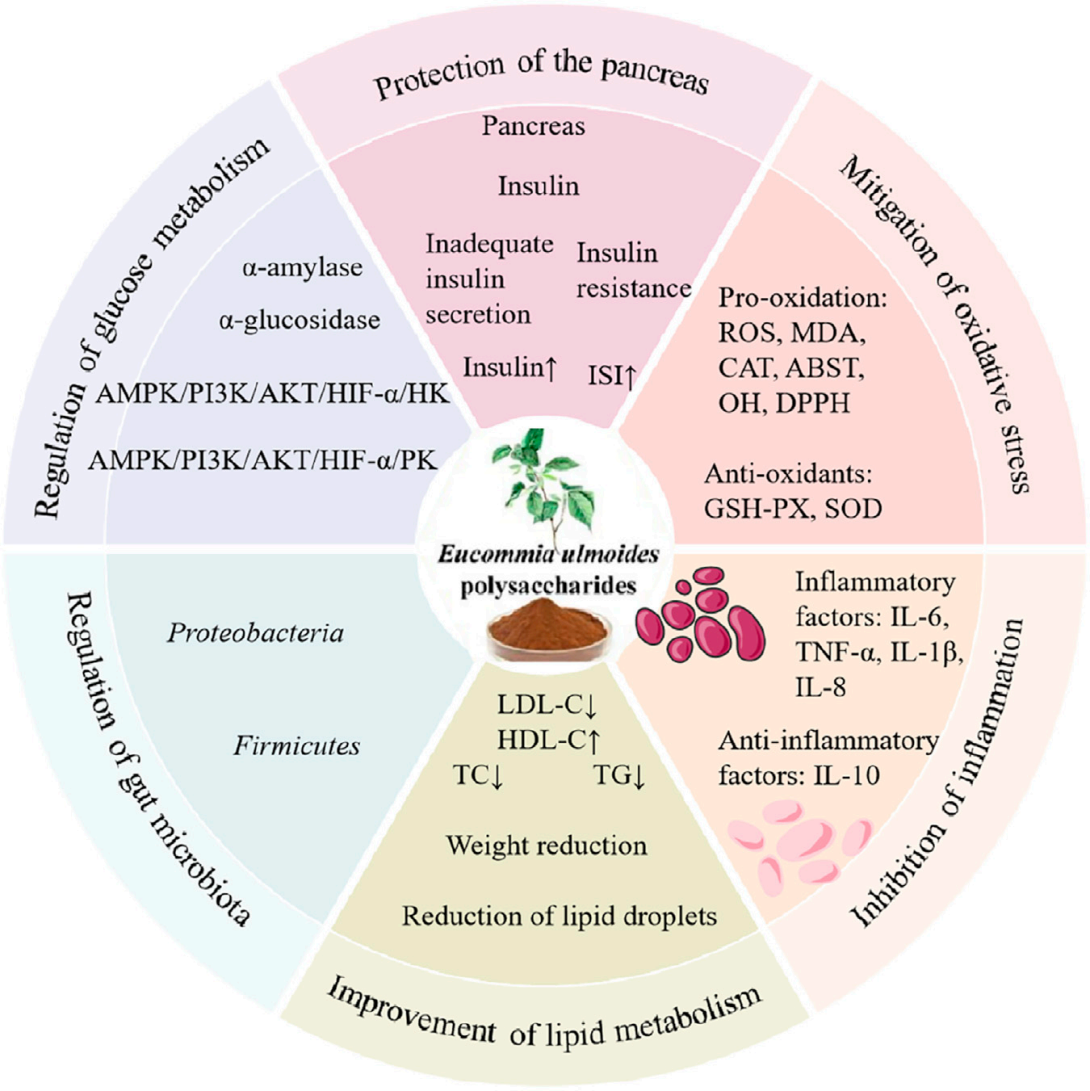
Pharmacological effects and mechanisms of EUP in the prevention and treatment of diabetes.

In conclusion, current experimental studies demonstrate that EUP exerts significant protective effects against diabetes through multiple mechanisms, including regulation of glucose metabolism-related gene and enzyme expression, inhibition of cellular apoptosis and protection of pancreatic β-cells, improvement of lipid metabolism disorders, modulation of oxidative stress, suppression of inflammatory responses; and regulation of gut microbiota composition (Figure 5).

### 3.7 Research gaps and future perspectives

While EUP’s hypoglycemic effects via AMPK/PPARγ pathways are documented, crosstalk between signaling cascades (e.g., PI3K/Akt, NF-κB) remains underexplored. Synergistic or antagonistic interactions necessitate validation via knockout models or multi-omics approaches. While EUP’s hypoglycemic effects via AMPK/PPARγ pathways are documented, crosstalk between signaling cascades (e.g., PI3K/Akt, NF-κB) remains underexplored. Synergistic or antagonistic interactions necessitate validation via knockout models or multi-omics approaches. While EUP’s hypoglycemic effects via AMPK/PPARγ pathways are documented, crosstalk between signaling cascades (e.g., PI3K/Akt, NF-κB) remains underexplored. Synergistic or antagonistic interactions necessitate validation via knockout models or multi-omics approaches (Zhang and Li, 2017; Zeng et al., 2018). While EUP’s hypoglycemic effects via AMPK/PPARγ pathways are documented, crosstalk between signaling cascades (e.g., PI3K/Akt, NF-κB) remains underexplored. Synergistic or antagonistic interactions necessitate validation via knockout models or multi-omics approaches.

## 4 Discussion and future directions

### 4.1 Current advances in polysaccharide-based health products

EU has attracted widespread attention for its health benefits in functional foods and medicine, owing to its status as a “medicinal food homologous” substance. Numerous health products have been developed using EU as a raw material, featuring diverse dosage forms such as beverages, teas, oral liquids, capsules, granules, and pills. These products demonstrate significant efficacy in relieving fatigue, enhancing immunity, lowering blood sugar and regulating lipid levels, increasing bone density, and improving sleep and bowel movements (Brugman et al., 2006). Wang et al. (2023) developed a novel health product, sweet rice wine incorporating EU leaf ultrafine powder, by adding the powder during glutinous rice fermentation. *In vitro* experiments confirmed this rice wine’s potent antioxidant, hypoglycemic, and lipid-lowering effects. This research validates the broader utilization of Eucommia ulmoides leaves, expands the variety of sweet rice wines, and further promotes EU’s health food market. Recent studies highlight that polysaccharides isolated and purified from EU bark and leaves have become a focal point of research due to their excellent bioactivity and high nutritional value. Consequently, the development of novel bioactive polysaccharides and polysaccharide-based functional foods and drugs constitutes a primary research focus.

### 4.2 Influence of extraction, isolation and purification methods on EUP structural characteristics

The structural features of polysaccharides, including glycosidic linkage types and branching patterns, can be significantly influenced by extraction methods. Different extraction techniques, due to their distinct mechanisms of action and operational conditions, may lead to polysaccharide chain cleavage, glycosidic bond conversion, or alterations in branching structures. Hot water extraction, as the most commonly used method, hot water extraction generally preserves the natural structure of polysaccharides due to its mild conditions. However, prolonged high-temperature treatment may result in partial degradation of polysaccharide chains, affecting the molecular weight distribution and degree of branching. Ultrasound accelerates the dissolution of polysaccharides through the cavitation effect, but high-intensity ultrasound may also cause polysaccharide chain cleavage, thereby impacting glycosidic linkage types and branching patterns. Therefore, optimizing ultrasound parameters (such as power and time) is crucial for maintaining polysaccharide structural integrity. Microwave extraction achieves instantaneous heating through electromagnetic fields, significantly improving extraction efficiency. However, the rapid heating process may lead to local changes in polysaccharide structure, such as glycosidic bond rearrangement or reduced branching structures. Alkaline treatment aids in the release of polysaccharides, but excessively strong alkaline conditions may disrupt glycosidic bonds, causing structural changes. Membrane filtration technology, especially ultrafiltration, separates polysaccharides with specific molecular weight ranges through membranes with different molecular weight cut-offs, thereby affecting the molecular weight distribution and branching degree of polysaccharides.

The purification process aims to remove impurities such as proteins, pigments, and inorganic salts from polysaccharides to obtain high-purity polysaccharides. However, the choice of purification method and operational conditions may also impact the structural characteristics of polysaccharides. Deproteinization methods, commonly used deproteinization methods include the Sevage method, TCA method, and HCl method. These methods remove proteins through different mechanisms but may also cause varying degrees of damage to polysaccharide structures. For example, while the HCl method exhibits high deproteinization efficiency, it readily leads to polysaccharide degradation. Therefore, when selecting a deproteinization method, it is necessary to strike a balance between deproteinization efficiency and polysaccharide structure preservation. H_2_O_2_ oxidation and activated carbon adsorption are commonly used decolorization methods. H_2_O_2_ oxidation exhibits high decolorization efficiency but requires further evaluation regarding its impact on polysaccharide structure. Activated carbon adsorption is relatively mild but the decolorization effect may be influenced by activated carbon type, dosage, and operational conditions. Ion-exchange chromatography and gel filtration chromatography are important methods for obtaining homogeneous polysaccharide components. These methods separate polysaccharides into components with specific molecular weight ranges, charge properties, and branching patterns through different separation mechanisms such as charge differences and molecular size exclusion. Thus, chromatographic purification not only aids in impurity removal but also reveals the structural diversity of polysaccharides.

Isolation and purification methodologies critically govern the structural attributes of EUP, including glycosidic connectivity, branching topology, and molecular weight. Strategic optimization of these processes is essential to preserve native architectures and isolate functionally relevant polysaccharide fractions. Future studies should establish quantitative structure-process correlations to advance industrial-scale EUP production for tailored applications.

### 4.3 Therapeutic potential of EUP in diabetes

Diabetes is now recognized as a “silent epidemic” that posing a significant threat to global health. Currently, natural products are attracting considerable research attention due to their low toxicity and minimal adverse effects, making them more suitable for long-term therapeutic use. Research on EUP, the primary active component of EU, for preventing and treating diabetes-related diseases remains in its early stages, with insufficient clinical studies and mechanistic investigations. Current research demonstrates that EUP possess multiple pharmacological activities, including blood sugar regulation, lipid reduction, antioxidant and anti-inflammatory properties, as well as gut microbiota modulation. These biological activities align with various pathophysiological mechanisms underlying diabetes-related complications, indicating eucommia polysaccharides’ potential as therapeutic candidates for diabetes management. The advancement of nanomedicine-based drug delivery systems has created novel opportunities for incorporating active components from TCM (Han et al., 2024). Future investigations should prioritize exploring eucommia polysaccharides as both therapeutic agents and drug delivery carriers to potentiate synergistic anti-diabetic effects.

Furthermore, structural modifications of EUP will be explored to enhance their anti-diabetic efficacy. Nevertheless, the clinical translation of EUP as novel therapeutic agents faces substantial challenges requiring systematic resolution. Critical research gaps encompass standardization of extraction and purification protocols, comprehensive structural characterization, and mechanistic understanding of structure-activity relationships. Consequently, future investigations should focus on elucidating precise chemical structures, establishing optimal dosage regimens and delivery routes, characterizing pharmacological profiles, and uncovering the material basis, molecular mechanisms, and therapeutic benefits against diabetes. Presently, EU research remains predominantly preclinical, with a striking paucity of clinical validation. This knowledge gap necessitates multidisciplinary research strategies integrating modern analytical techniques. With advancing quality control standards in TCM and growing public health consciousness, EUP-based therapeutics demonstrate considerable commercial viability in the nutraceutical and pharmaceutical markets.

## References

[B1] Ai X. P. Yu P. L. Li X. Y. Lai X. R. Yang M. Liu F. (2023). Polysaccharides from spirulina platensis: extraction methods, structural features and bioactivities diversity. Inter J. Biol. Macro. 231, 123211. 10.1016/j.ijbiomac.2023.123211 36632963

[B2] Bailey C. J. (2000). Potential new treatments for type 2 diabetes. Trends Pharmacol. Sci. 21 (7), 259–265. 10.1016/s0165-6147(00)01506-6 10871894

[B3] Bao L. Sun Y. Wang J. Li W. Liu J. Li T. (2024). A review of “plant gold” Eucommia ulmoides oliv.: a medicinal and food homologous plant with economic value and prospect. Heliyon 10 (2), e24851. 10.1016/j.heliyon.2024.e24851 38312592 PMC10834829

[B4] Berbudi A. Khairani S. Tjahjadi A. I. (2025). Interplay between insulin resistance and immune dysregulation in type 2 diabetes mellitus: implications for therapeutic interventions. Immunotargets Ther. 14, 359–382. 10.2147/ITT.S499605 40196377 PMC11974557

[B5] Brugman S. Klatter F. A. Visser J. T. Wildeboer-Veloo A. C. Harmsen H. J. Rozing J. (2006). Antibiotic treatment partially protects against type 1 diabetes in the bio-Breeding diabetes-prone rat. Is the gut flora involved in the development of type 1 diabetes? Diabetol 49/9, 2105–2108. 10.1007/s00125-006-0334-0 16816951

[B6] Chen X. H. Yang W. G. (2020). Optimization of ultrasonic assisted enzymatic extraction process for polysaccharides from Eucommia ulmoides leaves using response surface methodology. Food Ind. Sci. Techno 41 (22), 193–198. 10.13386/j.issn1002-0306.2020020298

[B7] Chen X. J. He F. G. Zhou D. Y. (2020). Effect of Eucommia ulmoides polysaccharide on db/db diabetic mice's glycolipid metabolism. Chin. Pharm. J. 55 (17), 1433–1438.

[B8] Chen Y. P. He J. P. Liu Y. Yang W. G. (2023). Optimization of ultrasonic microwave-assisted extraction process for polysaccharides from Eucommia ulmoides leaves and analysis of its in vitro anticoagulant activity. Food Ind. Sci. Techno 44 (17), 2022–2211. 10.13386/j.issn1002-0306.2022100189

[B9] Cui E. H. Tang P. Zhu X. Y. Lv M. Y. Wang S. Xue Y. H. (2023). Network pharmacology combined with an experimental validation study to reveal the effect and mechanism of Eucommia ulmoides leaf polysaccharide against immunomodulation. Foods 12 (5), 1062. 10.3390/foods12051062 36900578 PMC10001223

[B10] DeFronzo R. A. Ferrannini E. Groop L. Henry R. R. Herman W. H. Holst J. J. (2015). Type 2 diabetes mellitus. Nat. Rev. Dis. Prim. 11, 15019. 10.1038/nrdp.2015.19 27189025

[B11] Deng Y. Q. Ma F. B. Ruiz-Ortega L. I. Peng Y. Tian Y. He W. K. (2019). Fabrication of strontium Eucommia ulmoides polysaccharides and in vitro evaluation of their osteoimmunomodulatory property. Inter J. Biol. Macro 140, 727–735. 10.1016/j.ijbiomac.2019.08.145 31437498

[B12] Dludla P. V. Mabhida S. E. Ziqubu K. Nkambule B. B. Mazibuko-Mbeje S. E. Hanser S. (2023). Pancreatic β-cell dysfunction in type 2 diabetes: implications of inflammation and oxidative stress. World J. Diabetes 14 (3), 130–146. 10.4239/wjd.v14.i3.130 37035220 PMC10075035

[B13] Dworzański J. Strycharz-Dudziak M. Kliszczewska E. Kiełczykowska M. Dworzańska A. Drop B. (2020). Glutathione peroxidase (GPx) and superoxide dismutase (SOD) activity in patients with diabetes mellitus type 2 infected with epstein-barr virus. PLoS One 15 (3), e0230374. 10.1371/journal.pone.0230374 32210468 PMC7094858

[B14] Feng H. B. Fan J. Song Z. H. Du X. G. Chen Y. Wang J. H. (2016). Characterization and immunoenhancement activities of Eucommia ulmoides polysaccharides. Carbohydr. Poly. 136, 803–811. 10.1016/j.carbpol.2015.09.079 26572415

[B15] Gao Y. Zhang L. Zhang F. Liu R. Liu L. Li X. (2024). Traditional Chinese medicine and its active substances reduce vascular injury in diabetes via regulating autophagic activity. Front. Pharmacol. 15, 1355246. 10.3389/fphar.2024.1355246 38505420 PMC10949535

[B16] Gong P. Wang X. Han Y. Long H. Yang W. Chen F. (2024). Hypoglycemic activity of enzymatically extracted Eucommia ulmoides polysaccharide (EUL-w1) on IR-HepG2 cell via the AMPK/PI3K/Akt signaling pathway. Int. J. Biol. Macromol. 283, 137596. 10.1016/j.ijbiomac.2024.137596 39542294

[B17] Han J. Bao C. Duan J. (2024). Advances in the use of active components of traditional Chinese medicine as drug delivery nanocarriers. Chin. Her. Med. 55 (16), 5678–5691.

[B18] He X. R. Wang J. H. Li M. X. Hao D. J. Yang Y. Zhang C. L. (2014). Eucommia ulmoides oliv.: ethnopharmacology, phytochemistry and pharmacology of an important traditional Chinese medicine. J. Ethnopharmacol. 151 (1), 78–92. 10.1016/j.jep.2013.11.023 24296089

[B19] Hong Y. K. Liu W. J. Li T. She S. Y. (2013). Optimization of extraction of Eucommia ulmoides polysaccharides by response surface methodology. Carbohydr. Polym. 92 (2), 1761–1766. 10.1016/j.carbpol.2012.11.015 23399217

[B20] Huang W. Wang L. Lu M. (2014). Isolation, purification and structural characterization of an acidic polysaccharide fraction from leaves of Eucommia ulmoides oliver. named EOP-1. Fun Mate 4 (03), 3047–3050. 10.3969/j.issn.1001-9731.2014.03.011

[B21] Huang L. C. Lyu Q. Zheng W. Y. Yang Q. Cao G. (2021). Traditional application and modern pharmacological research of Eucommia ulmoides oliv. Chin. Med. 16/1, 73. 10.1186/s13020-021-00482-7 34362420 PMC8349065

[B22] Jeremiah S. S. Moin A. S. M. Butler A. E. (2024). Virus-induced diabetes mellitus: revisiting infection etiology in light of SARS-CoV-2. Metabolism 156, 155917. 10.1016/j.metabol.2024.155917 38642828

[B23] Ji J. L. Wang M. Liu G. P. (2021). Research on microwave assisted extraction of acidic polysaccharides and Eucommia ulmoides gum from Eucommia ulmoides seed shell. Liaoning Chem. Ind. 50 (4), 470–473.

[B24] Lang Q. Gong L. Ye J. Zhou Y. P. (2020). Hypoglycemic effect of the polysaccharide from Eucommia ulmoides leaves in diabetic rats. Mod. Food Sci. Technol. 36 (10), 27–32. 10.13982/j.mfst.1673-9078.2020.10.0388

[B25] Le X. N. Long D. P. Yin S. S. Qing R. Y. Chi Z. Z. Gao M. Q. (2025). The efficient separation of bioactive components from Eucommia ulmoides oliver using membrane filtration technology and its mechanisms in preventing alcoholic liver disease. Carbohydr. Polym. 351, 123100. 10.1016/j.carbpol.2024.123100 39779014

[B26] Li Q. Feng Y. He W. Wang L. T. Wang R. B. Dong L. (2017). Post-screening characterisation and in vivo evaluation of an anti-inflammatory polysaccharide fraction from Eucommia ulmoides. Carbohydr. Poly 169, 304–314. 10.1016/j.carbpol.2017.04.034 28504149

[B27] Liu G. R. Qiu Z. P. Zhou Y. M. Xin X. M. Gao Y. S. (2010). Effect and mechanism of EOP on diabetic mice induced by alloxan. J. Taishan Med. Coll. 31 (9), 659–661.

[B28] Liu C. Guo F. F. Xiao J. P. Wei J. Y. Tang L. Y. Yang H. J. (2020). Research advances in chemical constituents and pharmacological activities of different parts of eucommia ulmoides. J. Chin. Med. 45 (03), 497–512. 10.19540/j.cnki.cjcmm.20191108.201 32237506

[B29] Liu L. L. Zhang J. H. Cheng Y. Zhu M. Xiao Z. F. Ruan G. C. (2022). Gut microbiota: a new target for T_2_DM prevention and treatment. Front. Endocrinol. (Lausanne) 13, 958218. 10.3389/fendo.2022.958218 36034447 PMC9402911

[B30] Liu J. Li F. Yang L. Luo S. Deng Y. (2025). Gut microbiota and its metabolites regulate insulin resistance: traditional Chinese medicine insights for T2DM. Front. Microbiol. 16, 1554189. 10.3389/fmicb.2025.1554189 40177494 PMC11963813

[B31] Moradi K. Moghaddami R. Ghaffari-Nasab A. Khordadmehr M. Pagheh A. S. Mosajakhah H. (2025). Toxoplasma gondii modulates immune responses and mitigates type 1 diabetes progression in a streptozotocin-induced rat model. Cell Commun. Signal 23/1, 172. 10.1186/s12964-025-02168-1 40200271 PMC11980074

[B32] Peng Y. Yang Y. Tian Y. Zhang M. Cheng K. Zhang X. L. (2024). Extraction, characterization, and antioxidant activity of Eucommia ulmoides polysaccharides. Molecules 29, 4793. 10.3390/molecules29204793 39459162 PMC11510736

[B33] Qi X. Y. Zhou C. Y. (2011). Analysis of extraction process of Eucommia ulmoides polysaccharides by uniform design method. Chin. J. Exp. Pharm. 17 (13), 56–59. 10.13422/j.cnki.syfjx.2011.13.035

[B34] Qi S. H. Wu R. Jiang X. R. Fan G. Q. Wang Z. J. (2020). Optimization of flash extraction conditions for polysaccharides from EU leaves. Mod. Rural. Sci. Techno. 8, 84–85.

[B35] Ren N. Gong W. Zhao Y. Zhao D. G. Xu Y. (2023). Innovation in sweet rice wine with high antioxidant activity: eucommia ulmoides leaf sweet rice wine. Front. Nut 9, 1108843. 10.3389/fnut.2022.1108843 36704789 PMC9871602

[B36] Saeedi P. Petersohn I. Salpea P. Malanda B. Karuranga S. Unwin N. (2019). Global and regional diabetes prevalence estimates for 2019 and projections for 2030 and 2045: results from the international diabetes Federation diabetes atlas, 9^th^ edition. Diabetes Res. Clin. Pract. 157, 107843. 10.1016/j.diabres.2019.107843 31518657

[B37] Shen L. Chen X. Mu Y. J. Xu X. M. Liu B. B. Zhang K. (2023). Experimental study of eeucommia polysaccharide on HK-2 cell damage induced by high glucose. Chin. J. Diabetes 31 (2), 133–138.

[B38] Song J. Y. Zhang Y. F. Zhu Y. F. Jin X. Li L. Wang C. (2023). Structural characterization and anti-osteoporosis effects of polysaccharide purified from Eucommia ulmoides oliver cortex based on its modulation on bone metabolism. Carbohydr. Poly 306, 120601. 10.1016/j.carbpol.2023.120601 36746570

[B39] Su Z. Guo C. Liang T. (2016a). Effects of eucommiae cortex polysaccharide on streptozotocin-Induced diabetic mice. Chin. J. Exper Trad. Med. Form. 22 (14), 159–162. 10.13422/j.cnki.syfjx.2016140161

[B40] Su Z. Guo C. Liang T. (2016b). Effects of polysaccharides from Eucommia ulmoides on streptozotocin-induced diabetic mice. Chin. J. Exp. Form. 22 (14), 159–162.

[B41] Su J. Ren J. Chen H. Liu B. (2020). MicroRNA-140-5p ameliorates the high glucose-induced apoptosis and inflammation through suppressing TLR4/NF-κB signaling pathway in human renal tubular epithelial cells. Bios Rep. 40 (3), BSR20192384. 10.1042/BSR20192384 32073611 PMC7056448

[B42] Tian W. Huang J. Zhang W. Wang Y. Jin R. Guo H. (2024). Harnessing natural product polysaccharides against lung cancer and revisit its novel mechanism. Pharmacol. Res. 199, 107034. 10.1016/j.phrs.2023.107034 38070793

[B43] Tonin G. Dolžan V. Klen J. (2024). Genetic and transcriptomic background of oxidative stress and antioxidative therapies in late complications of type 2 diabetes mellitus: a systematic review. Anti (Basel) 13/3, 277. 10.3390/antiox13030277 38539811 PMC10967328

[B44] Wang Q. Y. He G. Z. Huang G. (2016). The effect of Eucommia ulmoides polysaccharides on serum SOD, GSH-Px, and MDA in diabetic model mice. Jiangxi J. Trad. Chin. Med. 47, 46–49.

[B45] Wang M. Sun P. Li Z. Li J. Lv X. Chen S. (2023). Eucommiae cortex polysaccharides attenuate gut microbiota dysbiosis and neuroinflammation in mice exposed to chronic unpredictable mild stress: beneficial in ameliorating depressive-like behaviors. J. Affect Dis. 334, 278–292. 10.1016/j.jad.2023.04.117 37156274

[B46] Wei J. J. Li X. J. Liu W. Chai X. J. Zhu X. Y. Sun P. H. (2023). Eucommia polysaccharides ameliorate aging-associated gut dysbiosis: a potential mechanism for life extension in drosophila. Inter J. Mole Sci. 24/6, 5881. 10.3390/ijms24065881 36982954 PMC10054339

[B47] Xia S. L. Pu J. (2019). Extraction of polysaccharides from Eucommia ulmoides leaves and their anti fatigue effects. Anhui Agricul Sci. 38 (33), 18747–18748. 10.13989/j.cnki.0517-6611.2010.33.011

[B48] Xu J. K. Hou H. J. Hu J. P. Liu B. C. (2018). Optimized microwave extraction, characterization and antioxidant capacity of biological polysaccharides from Eucommia ulmoides oliver leaf. Sci. Rep. 8 (1), 6561. 10.1038/s41598-018-24957-0 29700373 PMC5920044

[B49] Xu B. Q. Dai Y. Q. Fu Q. Y. (2020). Effects of Eucommia ulmoides polysaccharide on oxidative stress of pancreas in type 2 diabetic mice. Chin. Her. Med. 26 (10), 18–21. 10.13862/j.cnki.cn43-1446/r.2020.10.002

[B50] Xu S. Chen Y. Gong Y. (2024). Improvement of theaflavins on glucose and lipid metabolism in diabetes mellitus. Foods 13/11, 1763. 10.3390/foods13111763 38890991 PMC11171799

[B51] Yan Z. Q. Ding S. Y. Liu H. P. Chang M. L. Shi S. Y. Gong T. T. (2023). Optimization of extraction process and physicochemical characteristics of polysaccharides from Eucommia ulmoides leaves. J Tianjin univ sci techno. 38 (2), 11–18. 10.13364/j.issn.1672-6510.20220126

[B52] Yang S. M. Wang Z. J. Wang H. H. Zhang T. (2019). Ultrasonic assisted extraction and decolorization process of polysaccharides from Eucommia ulmoides bark. J. Dail Chem. Ind. 49 (7), 446–451.

[B53] Yang J. Wei H. Zhou Y. Szeto C. H. Li C. Lin Y. (2022). High-fat diet promotes colorectal tumorigenesis through modulating gut microbiota and metabolites. Gas 162 (1), 135–149.e2. 10.1053/j.gastro.2021.08.041 34461052

[B54] Zeng Q. Wei C. B. Xia F. Li X. (2018). Optimization of ultrasonic assisted extraction of chlorogenic acid from Eucommia ulmoides leaves and fuzhuan tea using response surface methodology and its in vitro hypoglycemic and antioxidant activities. Food Ferment Ind. 44 (09), 184–192. 10.13995/j.cnki.11-1802/ts.018104

[B55] Zhang T. T. (2020). Purification process of Eucommia ulmoides polysaccharides using macroporous resin and study on their resistance to exercise fatigue. Chem. Eng. 34 (4), 84–88. 10.16247/j.cnki.23-1171/tq.20200484

[B56] Zhang S. Li X. (2017). Advances in the study of the chemical composition and pharmacological effects of Eucommia ulmoides. Chin. J. Eth Folk. Med. 26 (10), 56–61.

[B57] Zhang X. J. Yi T. J. Sun Q. Y. Zhang W. K. Yan Y. P. Gong G. Z. (2011). Extraction, separation, complement-inhibitory activity, and structural study of polysaccharides from Eucommia ulmoides leaves. Nat. Prod. Res. Dev. 23 (04), 606–611. 10.16333/j.1001-6880.2011.04.028

[B58] Zhang L. Zhang H. Xie Q. Xiong S. Jin F. Zhou F. (2022). A bibliometric study of global trends in diabetes and gut flora research from 2011 to 2021. Front. Endocrinol. (Lausanne) 13, 990133. 10.3389/fendo.2022.990133 36339425 PMC9633665

[B59] Zhang J. X. Zhao J. Y. Liu G. Y. Li Y. D. Liang Li. Liu X. F. (2023). Advance in morchella sp. polysaccharides: isolation, structural characterization and structure-activity relationship: a review. Inter J. Biol. Macro 247, 125819. 10.1016/j.ijbiomac.2023.125819 37455001

[B60] Zhao B. Shangguan C. H. Yang H. Hu H. Z. Chen C. (2023). Analysis of development status of eucommiae cortex functional food. Chin. Trad. Herb. Drugs 54 (15), 5033–5043. 10.7501/j.issn.0253-2670.2023.15.028

[B61] Zhao X. Qu Q. Zhang Y. Zhao P. Qiu J. Zhang X. (2024a). Research progress of Eucommia ulmoides oliv and predictive analysis of quality markers based on network pharmacology. Curr. Pharm. Biotechno. 25 (7), 860–895. 10.2174/0113892010265000230928060645 38902931

[B62] Zhao L. Hu H. Zhang L. Liu Z. T. Huang Y. Liu Q. (2024b). Inflammation in diabetes complications: molecular mechanisms and therapeutic interventions. MedComm 5/4, e516. 10.1002/mco2.516 38617433 PMC11014467

[B63] Zheng J. L. Zhu Y. H. Zhang W. Sun G. T. Zhu M. Q. (2023). Life cycle assessment and techno-economic analysis of joint extraction of eucommia powder, gum, water-soluble polysaccharide and alkali-extractable polysaccharide from eucommia leaves. Process Biochem. 124, 235–244. 10.1016/j.procbio.2022.11.025

[B64] Zhu L. Hao X. L. Xu Y. Y. Tian X. X. (2022). Research on response surface optimization of Eucommia ulmoides polysaccharide extraction process. Agricul Techno Equip. 5, 64–66.

